# MONDE·T: A database and interactive webserver for non‐standard amino acids and peptide components in the PDB

**DOI:** 10.1002/pro.70809

**Published:** 2026-10-13

**Authors:** Alex Waldherr, Gesa L. Freimann, Andrei N. Lupas

**Affiliations:** ^1^ Department for Protein Evolution Max Planck Institute for Biology Tübingen Germany

**Keywords:** cell biology, database, non‐canonical amino acids, non‐standard amino acids, pharmaceutical optimization, post‐translational modifications, protein, protein design, protein structure, Ramachandran, synthetic biology, webserver

## Abstract

Non‐standard amino acids (ncAAs) together with synthetic additions (like caps and linkers) are of wide interest for protein design and pharmaceutical optimization, as they can improve physicochemical properties and possibly reshape protein structure. Their inclusion in experimental structures has not been explored systematically. It remains elusive which components are those most impactful for protein design. We therefore collected all non‐standard components present in the Protein Data Bank (PDB) into MONDE·T: A database of 1,210 unique ncAAs and 724 other synthetic components present in 23,149 PDB structures with a corresponding webserver. Analyses can focus on a single component or a single structure, illustrated here by example applications. MONDE·T allows for download of the data, visualization of a structure, identification of unusual backbone angles, and comparison of chemical similarities. The webserver is hosted at the Max Planck Institute Tübingen, accessible online via https://mondet.tuebingen.mpg.de/. MONDE·T is freely available under the Creative Commons 4.0 License.

## 1 INTRODUCTION

Non‐canonical amino acids (ncAAs) extend the repertoire of the genetically encoded canonical amino acids, both in the backbone and in the side chains. In the backbone, d‐amino acids introduce mirrored enantiomers of the standard set, while β‐amino acids introduce an additional carbon atom into the backbone, allowing for more flexible designs. The backbone can be further elongated by additional carbon atoms to yield γ‐, δ‐, ε‐, etc. amino acids. The backbone can also be elongated by the introduction of other elements between the amino and carboxylic groups.

As side chain modifications, nature already produces a wide range, both reversible and irreversible. For example, the most widespread by far is the reversible phosphorylation of serine, threonine, and tyrosine residues for cellular signal transduction (Fisher & Krebs, 1955; Worby & Dixon, 2019). Reversible modification of lysine residues in histone tails by acetylation and methylation also produces essential biological outcomes (Allfrey et al., 1964). Prominent irreversible modifications are the systematic conversion of prolines to hydroxyprolines in collagens (Bella et al., 1995), the *N*‐ and *O*‐linked glycosylation (Schellenberger, 1989; Varki et al., 2022) of side chains during the maturation of surface proteins, and the attachment of lipid tails to terminal cysteine residues (Olson, 1988) as membrane anchors. Beyond these, chemistry provides an almost endless list of synthetic modifications; for example, nucleotide bases can be used as side chains to produce PNA, peptide nucleic acids (Nielsen et al., 1991).

Such changes of chemical identity can influence the protein structure landscape in profound ways. For an example of backbone‐dependent structural effects, the structure and function of the ribosomally produced toxin polytheonamide B entirely depend on the systematic conversion of every second amino acid to the d‐form by a processive epimerase (Hamada et al., 2010).

Further extensive modifications of the side chains allow the toxin to fold into a membrane spanning ion channel that provides the toxic function (Parent et al., 2017). The same structure and toxicity can be brought about in gramicidin, which is produced by non‐ribosomal peptide synthases (Stachelhaus & Marahiel, 1995). For examples of side chain‐dependent structural effects, a new form of coiled coil not previously seen in nature could be generated by the systematic inclusion of the non‐canonical side chains alloisoleucine and norvaline (Harbury et al., 1998). The expectation in the field of protein design is that modified amino acids open entirely new areas of protein structure, both in the static and dynamic behavior of single proteins and their assemblies. The extent to which this expectation can be realized hinges critically on reaching an understanding of the interplay between backbone conformation and side chain packing preferences.

As a first step toward informing protein design with ncAAs, we prepared a catalogue of all their occurrences in experimentally verified structures. This catalogue primarily focuses on structure but will also prove useful for pharmaceutical design when ncAAs are included to diversify physicochemical properties and alleviate protease sensitivity often with the explicit intention not to change any structural properties. Previous efforts and chemical databases include the iNClusive database (Icking et al., 2024) (466 ncAAs), SwissSidechain (Gfeller et al., 2013) (288 ncAAs), and PSI‐MOD (Montecchi‐Palazzi et al., 2008) (615 ncAAs), which provide excellent starting points but lack the critical structural context and are incomplete in numbers compared to all global contributions made in the PDB. We found a surprisingly high count of ncAAs already included in experimental structures: 1,210 unique ncAAs (61‐times the canonical diversity) and 724 other backbone additions, present in 23,149 structures. This dataset was curated into MONDE·T and is now available as a database and webserver with tools for interactive analysis.

## 2 RESULTS

### 2.1 Data curation

To curate the MONDE·T dataset, we aimed to identify all cases in which ncAAs or fully synthetic caps were incorporated into a polypeptide chain in the Protein Data Bank (PDB). However, the PDB contains many other chain types in addition to polypeptide chains.

We therefore started from the worldwide chemical component dictionary (Berman et al., 2003) (CCD) (https://www.wwpdb.org/data/ccd, cut‐off date April 4, 2026) and included all present 50,001 components with their stereochemical SMILES (a string representation of their chemical graph) and global identifier (CCD ID, in the following all CCD IDs will be highlighted **bold**). This list presents an exhaustive list of all components registered for crystal structures, NMR structures and in cryo‐electron microscopy. Resultingly, many are small molecules (e.g., additives: glycerol **GOL**, ions: sodium **NA**) or ligands (pharmaceutical: aspirin **SAL**, or cofactors: Coenzyme A **COA**). We annotated these cases using the RCSB Search API (Rose et al., 2021) (https://search.rcsb.org) and removed them. Concretely, we queried how often a component appears as (i) unbound ligand, (ii) covalently bound, or (iii) incorporated in the polymer backbone and any ion, ligand or only covalently bound group that does not participate in the backbone was removed.

These true backbone components were further separated into backbone components occurring in polypeptides vs. those found in other chain types, like nucleic acids. In quantitative terms: We filtered the set of 50,001 unique components to a set of 2,811 true backbone components of which 1,933 are found within peptide backbones. Of these, 20 represent the canonical set, leaving 1,913 components which were included in MONDE·T. These include 1,210 ncAAs, their terminal derivates (e.g., amides), synthetic caps and linkers, and a set of autocatalytically formed GFP‐like chromophores subcategorized by substructure search. For each component, we addressed the RCSB Data API (Bittrich et al., 2023) (https://data.rcsb.org) and added further annotations. We annotated PDB IDs where the components occur, their functional description, the source organism, the experimental method, the experimental resolution. We added the standard one‐letter sequence (where any non‐standard component is denoted by **X**) and additionally prepared an unmasked version of each sequences where each component is presented by its CCD ID code instead (e.g., **AIB**). These are possibly useful for machine learning. Note that each CCD ID is usually three letters long but may occasionally contain five letters. Note further that for a given CCD ID, an overview page in PDB can be accessed under https://www.rcsb.org/ligand/XXX(XX).

The MONDE·T dataset is available under the Creative Commons 4.0 License. Filtered subsets or the whole annotated dataset can be copied to the clipboard or downloaded as CSV files via a prepared webserver.

### 2.2 Webserver

The webserver offers four functional areas, accessible from tabs at the top of the webpage. Figure 1 provides an overview of their functionalities.

**FIGURE 1:**
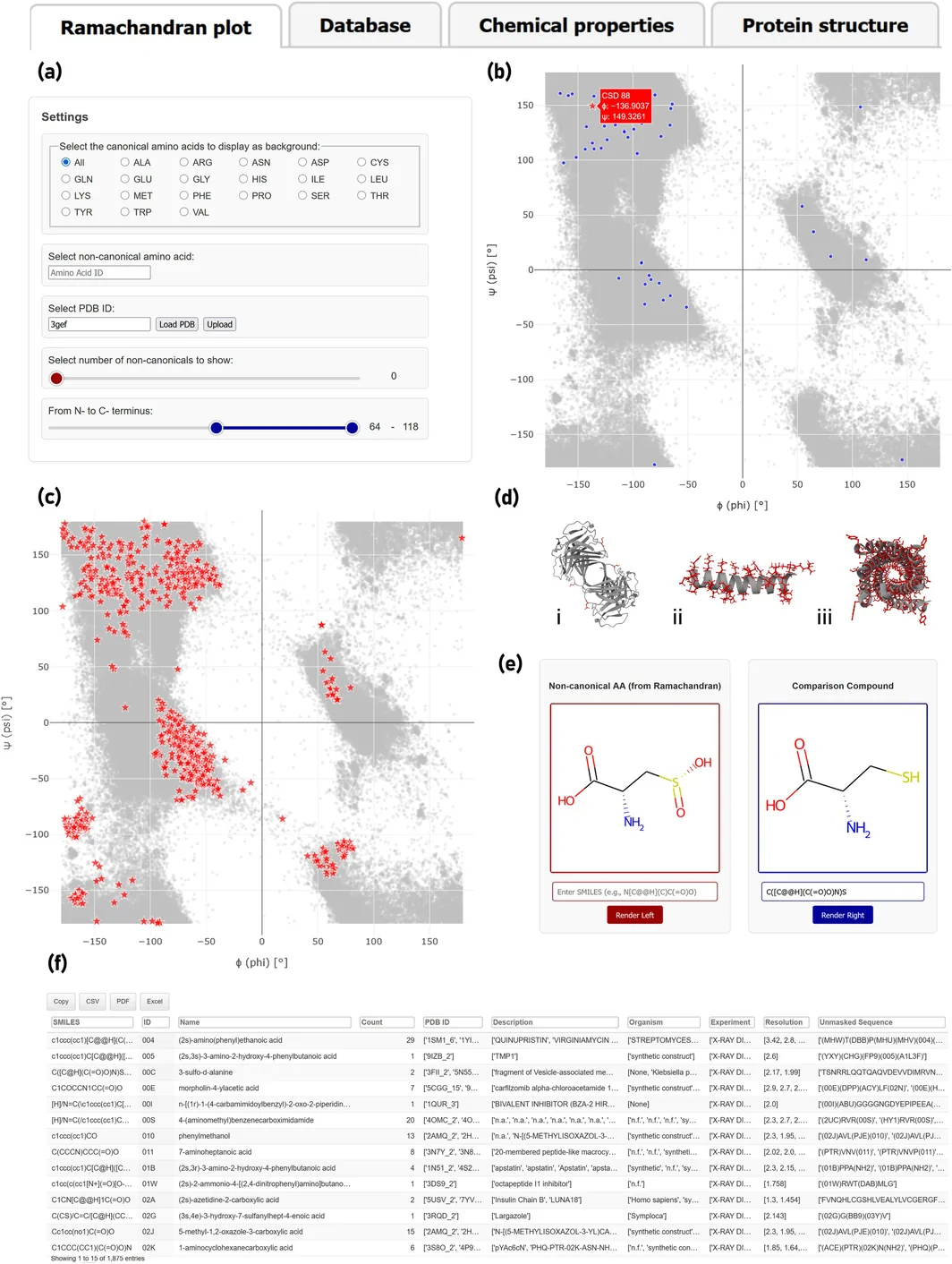
A collage of functionalities in MONDE·T: A user can explore 1D sequences, 2D Ramachandran plots, 3D structures and chemical properties. The webserver is split into four tabs. (a) Structural analysis starts in the *Ramachandran plot* tab. A PDB/MMCIF file (e.g., PDB ID 3GEF) can be uploaded or fetched (creates plot (b)). Alternatively, an ncAA of interest can be fetched via chemical component IDs (e.g., CCD ID **CSD**). Its metaplot of all occurrences in the PDB gets plotted (creates plot (c)). Upon hovering the Ramachandran plot displays more annotation. (d) If a protein is in focus, the fetched PDB ID prerenders the 3D structural view under the *Protein structure* tab. For speedy analysis, the ncAA residues are highlighted as red sticks. Shown in inserts: (i) PDB ID 3GEF (same structure as in plot (b)), (ii) PDB ID 2QOX (the toxin polytheonamide B), (iii) PDB ID 7QDI (the designed d‐AIB‐3_10_ helical bundle). (e) If an ncAA is in the focus of analysis, the compound of interest prerenders in the *Chemical properties* tab. This tab allows for comparison of SMILES strings and provides a catalogue of ncAAs per canonical parent. (f) The complete dataset can be searched and downloaded in the *Database* tab.

*Ramachandran plot tab*: A Ramachandran plot serves at the entry point to MONDE·T, because the backbone information provided by this plot type is central to understanding the structural properties of a polypeptide chain and its residues. Every residue in a polypeptide chain is described there by its angle ϕ to the preceding neighbor and its angle ψ to the succeeding neighbor. In the plot, the ϕ‐angle is shown on the *x*‐axis and the ψ‐angle on the *y*‐axis (Ramachandran et al., 1963). A user can analyze ncAAs or canonical proteins here.

To analyze an ncAA, a user enters a chemical component ID into the field “*Settings → Select non‐canonical amino acid*”. To analyze a protein structure, a user enters a PDB ID into the field “*Settings → Select PDB ID*” to fetch it from PDB or can upload a custom PDB/MMCIF file.

If a chemical component ID was entered, a scrollable table of all PDB entries, where this ncAA occurs, pops up. This table is interactive. By clicking on PDB entries in this table, the backbone angle for the ncAA in this entry is displayed as a red star on the Ramachandran plot to the right. To display the complete experimentally determined coverage for this ncAA, a slider can be dragged to count up the number of entries to display in the metaplot (be aware that terminal residues either lack a ϕ‐ or ψ‐angle and are therefore not plotted in the Ramachandran space).

To compare the ncAA's Ramachandran space to how canonical amino acids shape backbones, a selection of radio‐buttons in the field “*Settings → Select canonical background*” provides all canonical (ϕ, ψ)‐angles in PDB entries with a resolution ≤1.2 Å and *R*_observed_ ≤0.2 (1,511,165 residues in total).

If a PDB ID is entered or a PDB/MMCIF file was uploaded, the chains of the structure will be listed below the Ramachandran plot. By clicking on a chain, the user selects this chain and can build an interactive plot. Canonical residues are displayed as blue bullets. A region of interest can be set into focus with a blue slider on the left. More labels are displayed upon hovering, listing residue code and number. If an ncAA is present, it will be again marked as a red star. The curated plot can be “saved to disk” as PNG.

Since the analysis can be performed irrespective of whether an uploaded structure contains ncAAs, this tool offers rapid and intuitive access to the backbone geometry of any polypeptide chain.

*Database tab*: This tab provides access to the MONDE·T dataset and annotations. The table lists all chemical components with their CCD ID and more annotations. The table can be sorted by individual column through clicking on a column header or by entering a search term. Searches of individual columns can be combined to filter the dataset along two attributes. Of functional interest is the *Description* column, which provides approximate functional annotations as submitted by the PDB authors. The columns *PDB ID* and *Sequence* may also be of wide interest. The filtered or complete dataset can be downloaded to disk as a CSV file.

*Chemical properties tab*: This tab compares ncAAs to the 20 canonical amino acids. Two canvases are provided at the top. The left canvas is automatically populated (Landrum, 2006) with the chemical structure when an ncAA is fetched in the *Ramachandran plot* tab. Alternatively, a user can input any SMILES of interest and render their chemical structure to visually compare modifications. Below the canvases, a reference table with SMILES in MONDE·T and their Tanimoto similarity (Tanimoto, 1958) to the canonical components is given. Below the table, a set of catalogues can be opened. These catalogues show subsets of MONDE·T to reference all ncAAs belonging to a canonical parent, or on whether they are an l‐, d‐, β‐ or γ‐amino acid.

*Protein structure tab*: The final way to analyze ncAAs in polypeptide chains is to interactively explore 3D structures. This tab uses Mol* Viewer (Sehnal et al., 2021; Sun, 2023) (as in PDB) to display the structure uploaded in the *Ramachandran plot* tab in gray colors, with non‐canonical positions highlighted in red.

Below, application examples follow to exemplify possible workflows.

### 2.3 Application example: Posttranslational modifications

A widespread natural modification is the phosphorylation of residue side chains. If we wish to track structural examples of histidine phosphorylation (i) we query the *Database* tab with the search term *histidine* and (ii) obtain two IDs with a “‐phosphono‐” name prefix being 1‐phosphonohistidine (**HIP**, 16 entries) and 3‐phosphohistidine (**NEP**, 68 entries). (iii) These we fetch as ncAAs in the *Ramachandran plot* tab. We draw the top, dark red slider to visualize their backbone torsion angles against a selected canonical histidine background. (iv) We compare the two different phosphorylation sites (see Figure 2 top row) and observe that **NEP** spreads in the canonical histidine space, whereas **HIP** is concentrated at its periphery. (v) We further compare **HIP** to 1‐methylated histidine (**MHS**, 67 entries), methylated at the same position where **HIP** is phosphorylated. We find that **MHS** occupies a Ramachandran space that differs substantially from the canonical one.

**FIGURE 2:**
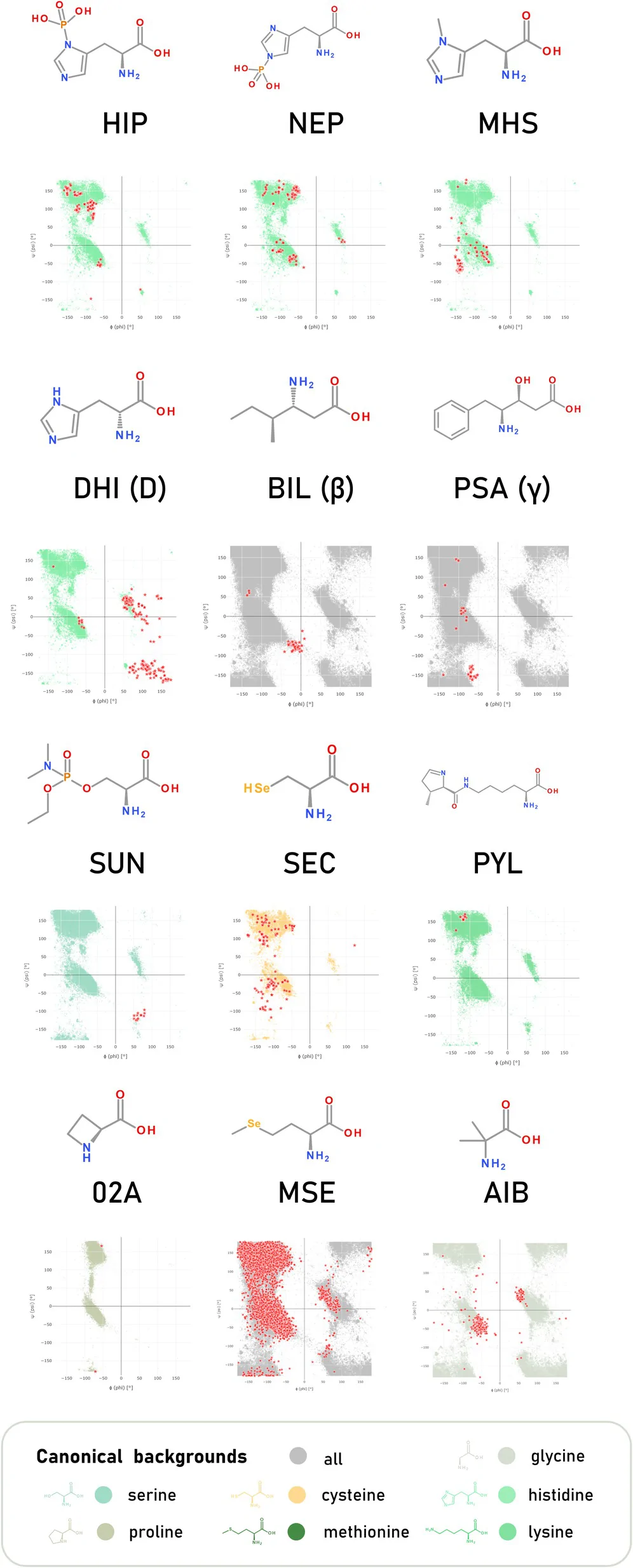
A collage of the ncAAs and their metaplots (mentioned in the application examples) in MONDE·T style. ncAAs are displayed as red stars over canonical backgrounds of choice. This allows for comparison of whether the ncAA fold landscape overlaps with canonical torsion angle distributions (gray = canonical background of the standard 20 amino acids).

We can in this way compare how the same modification acts at different positions, or how different modification types at the same position behave.

*Workflow*: *Database prefiltering → concrete identification of IDs and annotation data of interest → utilization of ID in the plotting modality → comparison of related chemical variants.*

### 2.4 Application example: Toxicology

Modification can also be forcefully introduced, for example by the toxin and serine modifier [dimethylamino(ethoxy)phosphoryl]‐formonitrile. This compound, notorious as the nerve agent tabun, acts by covalently modifying the catalytic serine in acetylcholinesterase. In MONDE·T, we can find it by (i) querying the column *Description* in the *Database* tab for “acetylcholinesterase” and (ii) querying the column *Name* for “dimethylamino.” We find the correct compound (**SUN**, 9 entries). We fetch **SUN** in the *Ramachandran plot* tab and can (iii) analyze a metaplot of its occurrences. We observe that the modified serine occupies an unusual Ramachandran space at the edge of the canonical distribution (Figure 2 center left). To also (iv) showcase how the fully canonical protein behaves, we fetch an unmodified acetylcholinesterase (e.g., PDB ID 2HA3) by entering this PDB ID into the *Load PDB* field. The catalytic serine (pos. 203) can be interactively explored: (v) We narrow down a focus region with the blue slider and (vi) hovering over the bullets. We find that the equivalent unmodified serine occupied an equally unusual space as **SUN**.

This exemplifies that specific folds can push both canonical amino acids and ncAAs into unusual spaces. The compound's own chemistry and its local context contribute to the shape of the backbone, and we ideally analyze both in tandem. The strongest change seen in backbone angles which are solely attributable to the chemistry of the component itself occurs for modification around the Cα‐carbon, see **DHI** (d‐amino acid), **BIL** (β‐amino acid), and **PSA** (γ‐amino acid) (see Figure 2 second row).

*Workflow*: *Database prefiltering → identification of IDs via Description and Name column → utilization of modified ID in the plotting modality → utilization of unmodified PDB ID as comparator control.*

### 2.5 Application example: Amino acid analogs

Biological systems have also evolved a number of amino acid analogs that are incorporated at the ribosome with toxic effects. For example, the toxin of lily‐of‐the‐valley flower, (2*S*)‐azetidine‐2‐carboxylic acid is a proline‐analog lacking one carbon in the ring (Fowden, 1956). It is misincorporated by the ribosome instead of proline and has teratogenic effects, severely affecting collagen structure (Joneja, 1981; Zagari et al., 1990). (i) A search for (2*S*)‐azetidine‐2‐carboxylic acid in the *Database* tab yields the abbreviation **02A** (2 entries) and (ii) its comparison with canonical proline in the *Ramachandran plot* tab shows **02A** at the edge of the proline distribution (see Figure 2 bottom left). No collagen structure incorporating **02A** is available in the PDB at present, obstructing toxicology research. This shows one open ncAA research gap.

Synthetically, the incorporation of amino acid analogs by mistranslation of sense codons can be pursued by selective pressure incorporation in which the organism is exposed to media with the ncAA analog replacing the canonical. The most widespread application by far is the incorporation of selenomethionine for crystallographic studies (Hendrickson et al., 1990). Correspondingly, the most abundant ncAA with least open gaps in MONDE·T is selenomethionine (**MSE**, 10,214 entries) which is used in place of methionine precisely because its structural properties are the same. Resultingly, the *Ramachandran plot* tab shows fully canonical distribution (Figure 2 bottom center), and **MSE** covers the methionine space completely.

### 2.6 Application example: Stop codon reassignment

Several ncAAs are introduced biologically not by posttranslational modification but by rededication of stop codons, for example selenocysteine (Leinfelder et al., 1988) (**SEC**, 93 entries) and pyrrolysine (Srinivasan et al., 2002) (**PYL**, 9 entries). These insertions are facilitated through highly specialized machinery directly at the ribosome. In the *Ramachandran plot* tab, we can (i) select cysteine or lysine backgrounds and (ii) plot the chemical IDs **SEC** or **PYL** across all their PDB occurrences against these (Figure 2 center right). We observe that **PYL** is fully contained within the canonical region, but **SEC** drifts into the non‐canonical space already seen for **MHS** above.

The same strategy of codon reassignment has been adopted by researchers to incorporate ncAAs for practical purposes. Limited by the effortful practice to introduce additional tRNAs and orthogonal synthetases into an organism to decode the suppressed stop codon, this method has been mostly restricted to functional studies and there are few structures in the PDB. One of the few examples, where 2‐cyanophenylalanine (**9IJ**, 16 entries) was included to probe the photosynthetic reaction center of *Rhodobacter sphaeroides* (PDB ID 8VTJ), shows complete occupancy of the ncAA residues inside the canonical Ramachandran space. This was verified by these authors to support their analysis of isolated functional effects (Tran et al., 2025).

In such cases, like with **MSE**, researchers specifically desire ncAAs which change a functional attribute but not the protein structure. This is equally true for pharmaceutical optimization. The *Chemical property* tab provides guidance and similarity scores for a palette of ncAAs to inform such efforts.

*Workflow*: *Known chemical component → fetch by chemical ID → plot and check Ramachandran landscape → optionally: compare against canonical background → find alternatives in the Chemical property tab → compare their chemical structures and verify extent of structural impact.*

### 2.7 Application example: Finding chemical candidates

If we were to find an ncAA for pharmaceutical optimization (i) we best start in the *Chemical properties* tab. (ii) At the bottom, bookmark tabs can be opened to select a parent amino acid and its catalogue of derivates. These catalogues span only derivates present in the PDB which guarantees that a structure, synthetic route or commercial option has been previously found. The count per derivate indicates its utilization. To find a pharmaceutically utilized non‐chiral derivate, we choose (iii) the glycine catalogue **GLY** (131 derivates) as parent and (iv) find α‐aminoisobutyric acid (**AIB**, 220 entries) with a utilization in 220 structures. We find these structures' PDB IDs (v) by fetching **AIB** in the *Ramachandran plot* tab. One PDB ID of pharmaceutical interest is 7KI0 of the peptide semaglutide (Lau et al., 2015; Zhang et al., 2021). We can (vi) go back to the *Chemical properties* tab where in the left canvas **AIB**'s chemical structure was now automatically prerendered. The user can (vii) use the Tanimoto similarity table to compare to the canonical set or (viii) copy any SMILES string of interest to compare **AIB** and render it in the right window. The user can also use own SMILES strings for a new, custom component not yet in MONDE·T or the PDB (Figure 3).

**FIGURE 3:**
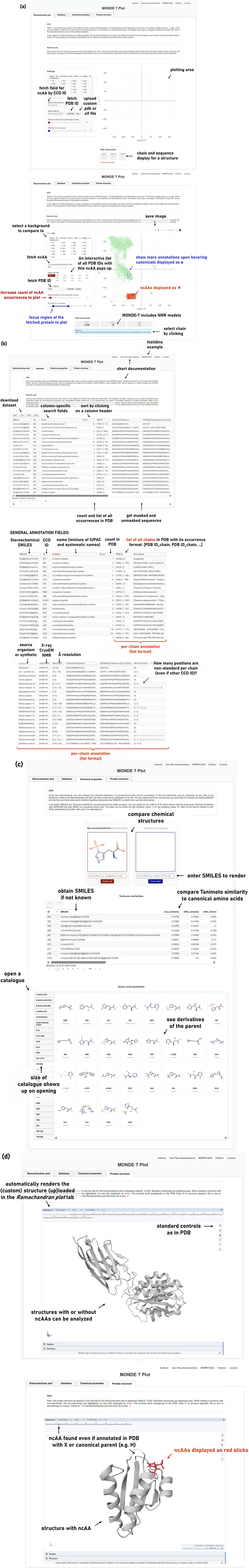
An overview of the tabs in MONDE·T and their functionalities with labels. (a) Entry page with the *Ramachandran plot*, where the plot in MONDE·T additionally serves as metaplot option to scan the landscape populated by a fetched ncAA. (b) *Database* tab. Column‐specific search and download buttons are the important functionalities. (c) *Chemical properties* tab. Top: Canvases to view and render SMILES into chemical structures. Bottom: Subset catalogues of MONDE·T which can be opened with chemical pictures. (d) *Protein structure* tab. Top: Default representation of a protein without canonical candidates. Bottom: Representation of a protein with ncAAs in MONDE·T style with red stick highlights.

*Workflow*: *Chemical property catalogues → visually identify an interesting candidate → find resolved PDB IDs in the Ramachandran plot tab → render SMILES and compare chemical graphs side‐to‐side.*

### 2.8 Application example: Chemical peptide synthesis

Evidently, chemical peptide synthesis is the method least inspired by a natural process or precursor. The incorporation of ncAAs by this method is fundamentally separated into two areas: site‐specific optimization of pharmaceutical candidates and global scaffold design for protein engineering. An example for site‐specific optimization was mentioned above with the exchange of residue by α‐aminoisobutyric acid (**AIB**, 220 entries) to prevent cleavage by dipeptidylprotease 4 in the currently widespread GLP‐1 agonist semaglutide (Lau et al., 2015; Zhang et al., 2021). Comparing the semaglutide structure (e.g., PDB ID 7KI0) in the *Ramachandran plot* tab against the canonical background shows its **AIB** in the canonical space of α‐helices.

Comparing the complete **AIB** landscape across all 220 occurrences in the PDB with the *Ramachandran plot* tab indicates a strong preference of **AIB** overall for both the l‐ and d‐α‐helical regions (Figure 2 bottom right). This is not equally reached by a canonical candidate as glycine is too flexible to stay contained within the α‐helical region, while the other amino acids prefer the l‐chiral α‐helical space.

Indeed, as a non‐chiral amino acid, the Ramachandran space of **AIB** is equivalent in l‐ and d‐configurations, making it very attractive for the design of new protein structures not accessible with the canonical amino acid set. Case in point, building coiled coils from 3_10_‐helices, rather than α‐helices, was only possible in d‐configuration with a third of the residues being **AIB** (PDB ID: 7QDI) (Kumar et al., 2022).

## 3 RECOMMENDATIONS FOR WORK WITH ncAAs

- Sodium is encoded as **NA** in the chemical component dictionary and is worthwhile to remove right at the start of any data analysis.
- Three‐letter codes are the standard for non‐canonical amino acids, yet, for example, derivates **A1IQX** or **A1AZ2** showcase this is not exclusively the case. 151 such five‐letter codes are in MONDE·T.
- Peptide nucleic acids (PNAs) exist though not included, as no agreement on their Ramachandran angles calculation exists; equally, β‐ and γ‐amino acids can cause futile angle calculations.
- No Ramachandran angle pairs can be defined for ncAAs at solely first or last position, for example, histidine amide HIA.
- Utilizing https://www.rcsb.org/ligand/XXX is useful to get an overview of whether a component is a ligand, covalently bound, or included in the true backbone. For example, **SAH** delivers worse predictability in RareFold, while checking here also shows that it is never a true backbone component.
- The PDB is influenced by reporting bias, and medically relevant proteins are predominant.
- The non‐ncAA components in MONDE·T include the set of 93 autocatalytically formed GFP‐like chromophores resolved in the PBD, components of interest for fluorescent protein design.
- *BioPython* can compute non‐canonical amino acid angles when the chain is set up via: *peptide = builder.build_peptides(structure, aa_only = 0)*

*Useful resources*: Other resources that list ncAAs outside structural context and may provide complementary value to readers are SwissSidechain (Gfeller et al., 2013), RESID (Garavelli, 2004), PSI‐MOD (Montecchi‐Palazzi et al., 2008), the CCD (Berman et al., 2003) and commercial repositories. SwissSidechain lists computational files to be used in molecular dynamics simulations, and plugins to be used in PyMol and UCSF Chimera for 230 components (Gfeller et al., 2013). RESID provides a list of 621 components, some annotated with their modifying enzyme (Garavelli, 2004). PSI‐MOD is a recently deprecated ontology of 615 ncAAs but the .obo file can still be accessed on their GitHub (Montecchi‐Palazzi et al., 2008). The chemical component dictionary is the gold standard for PDB ligand or component search. Commercially purchasable α‐amino acids are best summarized in the Enamine catalogue: https://enamine.net/building‐blocks/medchem/unnatural‐alpha‐amino‐acids.

*Expected growth*: As a rough growth estimate: Between April 2025 and April 2026, the wwPDB chemical component dictionary gained 3336 entries (+7%), of which MONDE·T gained 58 ncAAs (+5%), see Figure 4.

**FIGURE 4:**
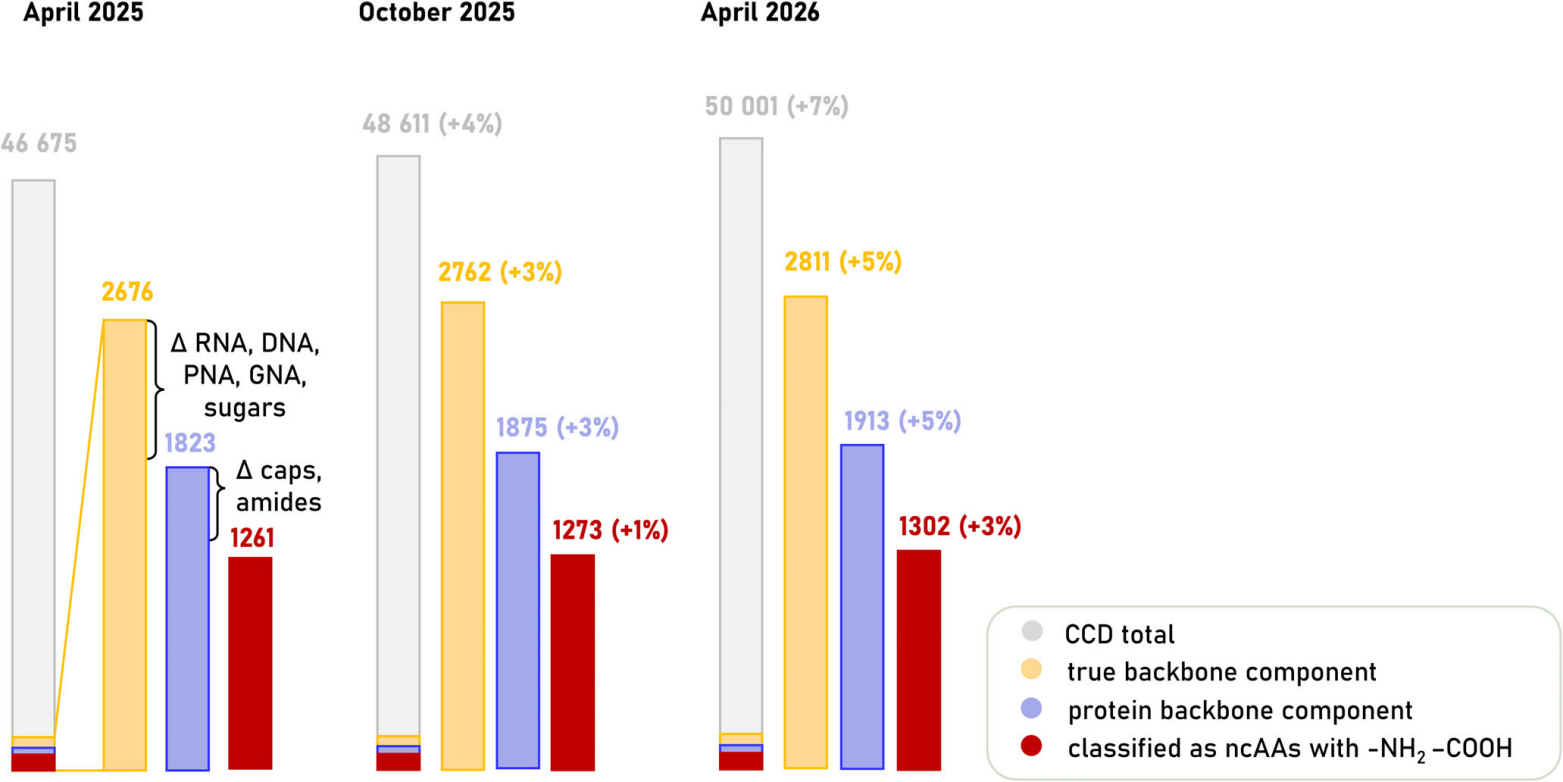
Annual growth of the wwPDB chemical component dictionary and the corresponding derived MONDE·T dataset in the year April 2025 to 2026. Gray: The total amount of chemical components registered in the PDB. Insert into the gray bar and zoom: Orange: Subset of the components truly participating in backbone formation (no unbound ligands, no covalent side chains only). Blue: Subset of these filtered backbone components, which are found in a polypeptide (no DNA/RNA components). This blue set comprises the set of components spanned by MONDE·T. Red: Subset of the components in MONDE·T which classify as non‐standard amino acid (ncAA).

## 4 SUMMARY

The MONDE·T toolkit provides a useful entry point for the exploration of ncAAs within polypeptide chains. The database is built entirely on proteins of known structure in the PDB. Our focus on experimental data makes MONDE·T an informative tool for application in protein design and pharmaceutical optimization. At the center of analyses performed through MONDE·T lies the Ramachandran plot of backbone torsion angles. These provide an instant 2D indicator of how components shape the protein backbones. The plot is usable for any polypeptide chain, whether or not it contains ncAAs, and can be adopted as a metaplot to show all occurrences of an ncAAs in PDB, providing rapid and interactive access to fold spaces in general. Users can explore 1D sequences, these 2D metaplots, 3D structures and chemical similarities. A *Database* tab allows users to download the data and obtain annotations. Access to MONDE·T is possible through chemical component IDs or PDB/MMCIF‐file which can be uploaded by the user or fetched from PDB. We anticipate that MONDE·T becomes a useful tool to scientists who consider the use of ncAAs for biomedical inquiry.

## AUTHOR CONTRIBUTIONS

**Alex Waldherr:** Conceptualization; investigation; writing – original draft; methodology; validation; visualization; writing – review and editing; software; data curation; project administration; formal analysis. **Gesa L. Freimann:** Software; project administration; visualization; methodology; writing – review and editing; conceptualization; formal analysis. **Andrei N. Lupas:** Funding acquisition; writing – review and editing; project administration; supervision; resources; formal analysis.

## CONFLICT OF INTEREST STATEMENT

The authors declare no competing interests.

## Data Availability

Data fully available as per the database and webserver submitted with this publication.
